# Eye health in farmers: The impact of environmental and occupational factors

**DOI:** 10.1371/journal.pone.0337944

**Published:** 2025-12-05

**Authors:** Athkar Alawneh, Mohammad Fraiwan, Fidaa Almomani

**Affiliations:** 1 Department of Natural Resources and Environment, Faculty of Agriculture, Jordan University of Science and Technology, Irbid, Jordan; 2 Faculty of Computer and Information Technology, Jordan University of Science and Technology, Irbid, Jordan; 3 Department of Rehabilitation Sciences, Jordan University of Science and Technology, Irbid, Jordan; West Bengal State University, INDIA

## Abstract

Farmers face higher health risks due to constant exposure to environmental and occupational hazards. Prolonged sun exposure increases the risk of UV-related eye damage (e.g., cataracts and pterygium), and frequent contact with dust, pesticides, and airborne debris can lead to chronic eye irritation, infections, and long-term vision problems. The physical demands of farm work, along with limited access to protective eyewear and healthcare, further compound these risks, and make eye health a significant concern in agricultural communities. This cross-sectional study examines the prevalence of eye ailments among farmers, exploring the environmental, occupational, and demographic factors contributing to these conditions. Data were collected from 893 participants and analyzed using a descriptive-correlational methodology to identify associations affecting farmers’ eye health. Individuals whose primary or secondary profession is agriculture reported a greater impact of climate on their eye health (p=0.003), while farmers experienced a more significant effect of agricultural activities on their vision (p=0.007), despite being more likely to use protective equipment. However, no meaningful correlation was found between farming and the prevalence of specific eye illnesses. These findings emphasize that farmers are particularly vulnerable to environmental and occupational factors that negatively impact the daily eye health. Despite their increased use of protective equipment, they still reported significant effects from climate conditions and agricultural activities. This suggests that current protective measures may be insufficient or. inconsistently used. While no direct correlation was found between farming and ipecific eye illnesses, the cumulative impact of environmental exposure on vision health remains a concern. Hence, there is a need for improved protective strategies, increased access to eye care, and targeted interventions to mitigate the occupational risks faced by farmers.

## Introduction

Farming activities entail the cultivation of crops and the raising of livestock for food, fiber, and other agricultural products. Farmers are uniquely vulnerable to eye diseases due to their constant exposure to UV radiation [1,2], airborne particulates, pesticides [3], and mechanical hazards . They are disproportionately exposed to environmental and occupational hazards that elevate their risk of developing eye diseases [4]. These risks are compounded by limited protective measures, inadequate healthcare access, and environmental extremes [5]. Extreme weather conditions further compound the risks faced by farmers. High temperatures and prolonged sun exposure contribute to corneal dehydration, intensifying symptoms of dry eye, while cold temperatures and strong winds can lead to excessive tearing and corneal sensitivity [6,7]. Seasonal changes, particularly in regions with significant temperature fluctuations [8], exacerbate ocular discomfort and increase the likelihood of developing long-term eye conditions [9].

Farmers experience prolonged exposure to UV radiation, which has been linked to conditions such as cataracts, pterygium, and photokeratitis [10]. UV rays cause oxidative damage to the lens and cornea, accelerating the formation of cataracts, a leading cause of blindness worldwide [11]. Additionally, wind, dust, and dry air exacerbate ocular surface conditions like dry eye syndrome and chronic conjunctivitis, particularly in arid and semi-arid farming regions [12]. Seasonal variations, including extreme temperatures, further contribute to corneal irritation and inflammation. Farmers working in highly reflective environments, such as sandy or snow-covered fields, face an even higher risk of developing photokeratitis, a painful condition often described as sunburn of the eye. Although UV-blocking sunglasses or protective eyewear can mitigate these effects, many farmers do not use them regularly due to discomfort, cost, or lack of awareness [13].

Agricultural workers frequently handle pesticides, herbicides, and fertilizers, many of which contain toxic chemicals that are harmful to ocular tissues. Exposure to organophosphates and carbamates has been associated with optic neuropathy, retinal damage, and corneal burns [14]. Studies indicate that pesticide exposure significantly increases the risk of chronic eye irritation, uveitis, and retinal degeneration [15]. Furthermore, airborne irritants such as dust, smoke, and plant debris cause allergic conjunctivitis, corneal abrasions, and chronic eye infections [16]. Farmers who operate machinery or work near plowing fields are at heightened risk due to the high concentration of airborne debris. Corneal irritation and inflammation are common consequences, as small dust particles can cause micro-abrasions that, if left untreated, may lead to infection or long-term damage [17].

Despite the recognized risks, many farmers either lack access to protective eyewear or do not use it consistently due to discomfort, cost, or lack of awareness [13]. Studies have shown that even when protective measures are available, farmers underutilize them, leading to sustained exposure to hazardous conditions [18]. Moreover, access to healthcare remains another significant challenge for farmers. Many work in remote or rural areas where eye care services are scarce, leading to delayed diagnoses and treatment of ocular conditions. Financial limitations further prevent regular eye check-ups, and the lack of specialized ophthalmological services in rural settings means that many farmers endure vision problems without timely medical intervention. As a result, minor eye conditions often progress into more severe impairments, ultimately affecting their quality of life and productivity.

## Materials and methods

### Ethical approval declarations

The study was conducted in accordance with the Declaration of Helsinki, and approved by the Institutional Review Board of King Abdullah University Hospital and the Deanship of Research, at Jordan University of Science and Technology, Jordan (Ref. 99/301/2024 Date 30/11/2024). Written informed consent was collected from all participants before they completed the questionnaire.

### Design

This study employed a cross-sectional design with a descriptive-correlational methodology to investigate the prevalence and risk factors associated with eye health as a result of practicing farming.

### Participants

Participants were randomly recruited from the local community between December 15, 2024, and April 15, 2025. It included a representative sample of farmers, casual farmers and non-farmers. To determine the necessary sample size, a power analysis was conducted using the Cochran’s formula for finite populations, see Eq 1. Thus, the necessary sample size for a population of 350,000 (i.e., number of individual associated with agriculture in Jordan [19]) with a 95% confidence level and a 5% margin of error is approximately 384 respondents.

$$n_{finite}=\frac{nN}{N+n-1}$$ (1)

Where:

$n=\frac{Z^{2}p(1-p)}{e^{2}}$

N = 350,000 (population size)

Z = 1.96 (Z-score for 95% confidence level)

p = 0.5 (proportion, set to 0.5 for maximum variability)

e = 0.05 (margin of error, 5%)

### Procedure

The questionnaire gathered socio-demographic data, including whether they identify themselves as agricultural workers (primary or secondary) or not, age, gender, income, and educational level, as well as work-related details such as income, work sector, nature of work (office vs field), number of working days per week, and time of work (night or day). It also investigated factors potentially correlated with eye diseases in farmers, which included frequency of visits to an eye doctor and prior knowledge of climate change effects. The study involved four outcome variables, including the effect of the environmental/weather factors on eye health, having an eye illness, effect of agricultural activities on eye health, and the use of protective equipment. These factors were calculated based on several questions as shown in Table 1.

**Table 1:** The questions associated with each outcome variable.

Outcome Variable	Question
Effect of the environmental/weather factors on eye health	Do you suffer from your eye problems in dry or wet weather conditions?
	When the wind speed increases, do you have eye problems
	In cold weather do you suffer from eye problems?
	When the temperature rises, do you suffer from eye problems?
	When it rains, do you suffer from eye problems?
	In dusty weather do you suffer from eye problems?
	When exposed to sunlight, do you suffer from eye problems?
Having an eye illness	Do you suffer from dry eyes?
	Do you suffer from burning eyes?
	Do you suffer from red eyes?
	Do you suffer from blurred vision?
	Do you suffer from itchy eyes?
	Do you experience sensitivity to light?
	Do you suffer from pimples inside your eyes?
	Do you have difficulty seeing at night?
	Do you suffer from headaches?
	Do you suffer from severe pain in your eyes?
	Do you suffer from lack of tear production?
Effect of agricultural activities on eye health	When picking fruits, do you get pollen in your eyes?
	When digging to plant trees, do you risk getting soil particles into your eyes?
	Do you suffer from eye allergy to any fruit?
	When spraying pesticides, do you suffer from eye injuries?
	When digging the soil, do you suffer from eye injuries?
	When pruning trees, do you suffer from eye injuries?
	When watering the field, do you suffer from eye problems?
Use of protective equipment	Do you use prescription glasses?
	Do you use sunglasses?
	Do you use eye protection?
	Do you use eye protection while practicing agriculture?

In the survey, some questions had four response levels, while others had three. During the statistical analysis, a decision was made to reduce the number of levels for certain questions in cases where either “sometimes” or “rarely” had very few responses. To ensure sufficient statistical power, these two intermediate categories were merged, and this adjustment was accounted for in the analysis. This modification affected four questions related to eye illnesses presented in Table 6, all of which showed insignificant or very weak associations with being a farmer. It is important to note that participants were generally able to distinguish between “sometimes” and “rarely” in the context of agricultural activities. Specifically, “rarely” referred to occurrences happening only a few times during an agricultural season (e.g., once or twice when performing tasks such as digging or pruning), whereas “sometimes” described problems that occurred more regularly but not consistently (e.g., several times during the season, though not every time the task was performed). While participants’ interpretations may naturally vary, including both categories helped capture meaningful differences in the frequency of eye-related issues during agricultural work.

### Data processing and statistical analyses

**Fig 1:**
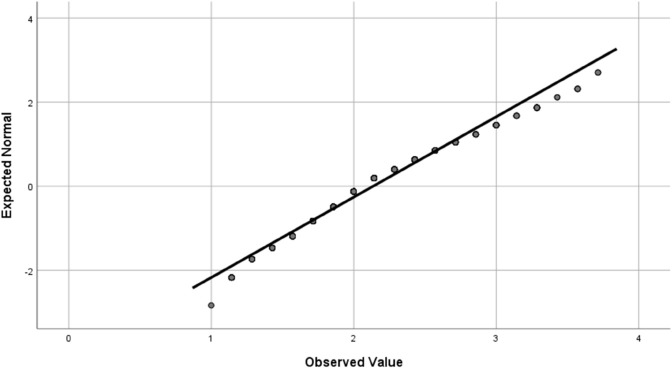
The Q-Q plot for the effect of the environmental factors on eye health outcome variable confirming the normal distribution of the score.

**Fig 2:**
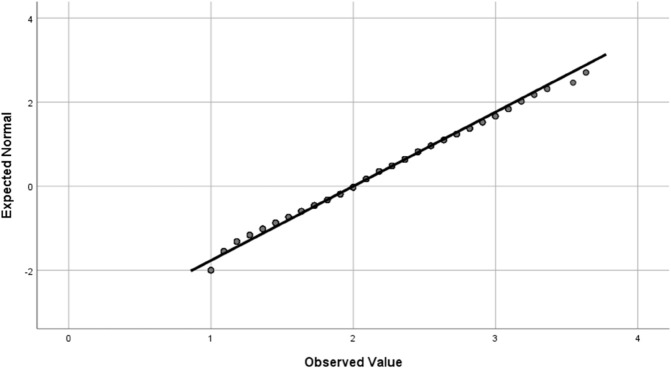
The Q-Q plot for the (having eye illness) outcome variable confirming the normal distribution of the score.

**Fig 3:**
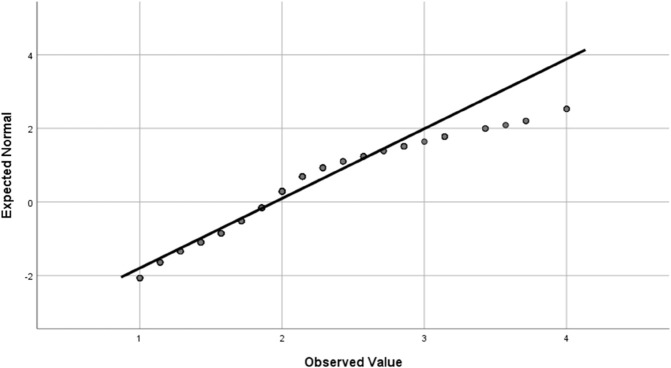
The Q-Q plot for the effect of agricultural activities on eye health outcome variable confirming the normal distribution of the score.

**Fig 4:**
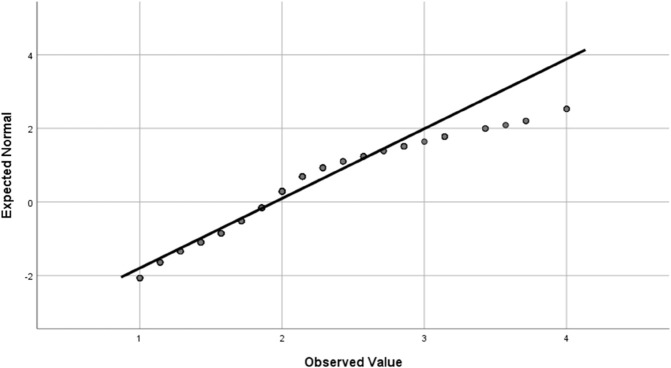
The Q-Q plot for the use of protective equipment outcome variable confirming the normal distribution of the score.

Data analysis was conducted using SPSS statistical software statistical summaries were generated for the dependent variable and key factors. For independent categorical variables, a chi-square test was used to compare frequencies between two groups, and a one-way analysis of variance (ANOVA) was applied for variables with more than two groups. Fisher’s least significant difference (LSD) test was then used to interpret significant differences (i.e., *p*<0.05). Four outcome variable were studied; the effect of the environment on farmers’ eye health, having eye illness, effect of agricultural activities on eye health, and whether the subject uses sunglasses and eye protection. The statistical methods required that these outcome variable are normally distributed, which was verified using quantile-quantile (Q-Q) plot, as shown in Figs 1, 2, 3, and 4.

Once the significant predictors were identified for each outcome variable, multiple linear regression was conducted to generate more robust findings by evaluating the unique contribution of each factor while controlling for the influence of others. Both standardized and unstandardized coefficients of the fitted models were reported to aid interpretation. Multicollinearity among predictors was assessed using the variance inflation factor (VIF), which is the reciprocal of tolerance. According to the literature, a VIF of 1 indicates no multicollinearity, values between 5 and 10 suggest potential concerns, and values above 10 reflect serious multicollinearity problems [20].

## Results

Table 2 presents demographic and occupational characteristics of the sample population (N = 893) based on their primary or secondary engagement in agriculture or non-farming status. Males (43.7%) are more likely to have agriculture as their main profession (63.1%), whereas females (56.3%) are more prevalent among those who are not farmers (68.8%). The largest age group is 18-23 years (47.0%), with most of them not being farmers (56.0%). The majority earn less than 500 JOD (45.4%), while only 2.9% earn more than 2001 JOD. Most respondents visit an eye doctor every five years (44.2%), while 3.6% go monthly, and 7.8% never visited one. Education levels are high, with 67.7% holding a bachelor’s degree, while only 2.4% have a basic education. Government employment (58.8%) is more common than private sector work (41.2%). A majority work five days a week (41.7%), with 13.5% working daily. Most (76.9%) work during the day, while only 4.8% work exclusively in the evening. Climate change awareness is high (66.3%), though 23% are uncertain, and 10.8% are unaware of it.

**Table 2:** Frequency and percentages of demographic variables. 1 USD = 0.71 JOD (fixed rate).

Variable	Level	Primarily a farmer	Secondary profession is farming	Not a farmer
Gender	Male	65 (63.1%)	181 (55.0%)	144 (31.2%)
	Female	38 (36.9%)	148 (45.0%)	317 (68.8%)
Age (years)	18-23	31 (30.1%)	131 (39.8%)	258 (56.0%)
	24-29	23 (22.3%)	43 (13.1%)	62 (13.4%)
	30-35	14 (13.6%)	20 (6.1%)	22 (4.8%)
	36-41	6 (5.8%)	31 (9.4%)	21 (4.6%)
	42-50	19 (18.4%)	59 (17.9%)	63 (13.7%)
	>50	10 (9.7%)	45 (13.7%)	35 (7.6%)
Income (JOD )	< 500	57 (55.3%)	163 (49.5%)	185 (40.1%)
	501–1000	38 (36.9%)	138 (41.9%)	205 (44.5%)
	1001–2000	6 (5.8%)	23 (7.0%)	52 (11.3%)
	>2001	2 (1.9%)	5 (1.5%)	19 (4.1%)
I visit an eye doctor every	Five years	35 (34.0%)	133 (40.4%)	227 (49.2%)
	Four years	8 (7.8%)	15 (4.6%)	14 (3.0%)
	Three years	20 (19.4%)	78 (23.7%)	101 (21.9%)
	Two years	8 (7.8%)	46 (14.0%)	53 (11.5%)
	Year	11 (10.7%)	24 (7.3%)	18 (3.9%)
	Monthly	7 (6.8%)	10 (3.0%)	15 (3.3%)
	Never	14 (13.5%)	23 (7%)	33 (7.2%)
Educational level	Basic stage	8 (7.8%)	10 (3.0%)	3 (0.7%)
	Secondary	20 (19.4%)	56 (17.0%)	39 (8.5%)
	Diploma	11 (10.7%)	34 (10.3%)	42 (9.1%)
	BSc	55 (53.4%)	199 (60.5%)	351 (76.1%)
	Masters or PhD	9 (8.7%)	30 (9.1%)	26 (5.6%)
Work sector	Governmental	52 (50.5%)	195 (59.3%)	278 (60.3%)
	Private	51 (49.5%)	134 (40.7%)	183 (39.7%)
Nature of work	Office	24 (23.3%)	101 (30.7%)	172 (37.3%)
	Field	44 (42.7%)	142 (43.2%)	150 (32.5%)
	Office and field	35 (34.0%)	86 (26.1%)	139 (30.2%)
Number of working days per week	One day	1 (1.0%)	6 (1.8%)	20 (4.3%)
	Two days	4 (3.9%)	14 (4.3%)	23 (5.0%)
	Three days	11 (10.7%)	36 (10.9%)	60 (13.0%)
	Four days	8 (7.8%)	43 (13.1%)	62 (13.4%)
	Five days	36 (35.0%)	144 (43.8%)	192 (41.6%)
	Six days	25 (24.3%)	39 (11.9%)	48 (10.4%)
	Daily	18 (17.5%)	47 (14.3%)	56 (12.1%)
Time of work	Daytime	68 (66.0%)	252 (76.6%)	367 (79.6%)
	Evening	10 (9.7%)	18 (5.5%)	15 (3.3%)
	All times	25 (24.3%)	59 (17.9%)	79 (17.1%)
Do you have prior knowledge of climate change?	Yes	71 (68.9%)	233 (70.8%)	288 (62.5%)
	No	15 (14.6%)	31 (9.4%)	50 (10.8%)
	Maybe	17 (16.5%)	65 (19.8%)	123 (26.7%)

Table 3 summarizes the association between environmental/weather factors and eye health across professional groups in agriculture. Significant effects were observed for rising temperatures ($\chi^{2}(4,893)$=13.789, p=0.008), where non-farmers reported more symptoms than farmers, and for rainfall ($\chi^{2}(4,893)$=30.235, p=0.000), with non-farmers more often reporting no symptoms. Dusty weather also showed a significant association ($\chi^{2}(4,893)$=10.141, p=0.038). By contrast, wind ($\chi^{2}(4,893)$=6.740, p=0.150) and sunlight ($\chi^{2}(4,893)$=9.241, p=0.055) were not significantly linked to symptoms, while cold weather showed only borderline significance ($\chi^{2}(4,893)$=12.585, p=0.050). The analysis shows that dryness, rainfall, and heat emerged as the most impactful environmental factors, with differences between farmers and non-farmers suggesting variation in exposure or adaptation.

**Table 3:** The Chi-square test significance results for the environmental factors effect on eye health among subjects with different professional engagements in agriculture. **: statistically significant with p < .01.

Do you suffer from eye problems in ?	Level	Primarily a farmer	Secondary profession is farming	Not a farmer	$\chi^{2}(4,893)$	P
high wind speed	Never	12 (11.7%)	24 (7.3%)	47 (10.2%)	6.740	.150
	Rarely	66 (64.1%)	249 (75.7%)	337 (73.1%)		
	Always	25 (24.3%)	56 (17.0%)	77 (16.7%)		
cold weather	Never	25 (24.3%)	86 (26.1%)	137 (29.7%)	12.585	.050
	Rarely	25 (24.3%)	109 (33.1%)	146 (31.7%)		
	Sometimes	39 (37.9%)	111 (33.7%)	154 (33.4%)		
	Always	14 (13.6%)	23 (7.0%)	24 (5.2%)		
high temperatures	Never	27 (26.2%)	70 (21.3%)	138 (29.9%)	13.789	.008**
	Rarely	57 (55.3%)	219 (66.6%)	280 (60.7%)		
	Always	19 (18.4%)	40 (12.2%)	43 (9.3%)		
rainy weather	Never	38 (36.9%)	143 (43.5%)	254 (55.1%)	30.235	.000**
	Rarely	53 (51.5%)	175 (53.2%)	193 (41.9%)		
	Always	12 (11.7%)	11 (3.3%)	14 (3.0%)		
dusty weather	Never	7 (6.8%)	16 (4.9%)	49 (10.6%)	10.141	.038
	Rarely	64 (62.1%)	206 (62.6%)	287 (62.3%)		
	Always	32 (31.1%)	107 (32.5%)	125 (27.1%)		
exposure to sunlight	Never	15 (14.6%)	40 (12.2%)	78 (16.9%)	9.241	.055
	Rarely	60 (58.3%)	220 (66.9%)	306 (66.4%)		
	Always	28 (27.2%)	69 (21.0%)	77 (16.7%)		

Multiple linear regression was used to test if the environmental factors effect on eye health was explained by the significant variables in Table 3. The regression was statistically significant (*R*^2^ = 0.905, F(4,890) = 1691.116, p < 0.001), see Tables 4 and 5. This indicates that the variables explain 90.5% of the variance in the outcome variable. Furthermore, Multicollinearity among the variables was assessed using the VIF. All VIF values were between 1 and 1.51, which indicates a high level of acceptability.

**Table 4:** Linear regression model summary and ANOVA results. ^a^ Predictors are the significant factors in Table 3.

Model	*R*	*R* ^*2*^	Adjusted *R*^*2*^	Std. Error of the Estimate
1	.951^a^	.905	.904	.15999

**Table 5:** Regression ANOVA results. Dependent variable is the effect of the environmental factors on eye health. Predictors same as Table 4.

Model	Sum of squares	df	Mean square	F	Sig.
Regression	216.442	4	43.288	1691.116	.000
Residual	22.782	890	.026		
Total	239.224	894			

Table 6 presents the distribution of eye-related problems across professional groups. Most conditions, including dry eyes, burning eyes, blurred vision, itchy eyes, and sensitivity to light, showed no significant association with profession. In contrast, significant differences were observed for red eyes ($\chi^{2}(6,893)$=22.687, p=.001), pimples inside the eyes ($\chi^{2}(6,893)$=35.629, p=.000), difficulty seeing at night ($\chi^{2}(6,893)$=14.581, p=.024), headaches ($\chi^{2}(6,893)$=10.694, p=.030), severe eye pain ($\chi^{2}(6,893)$=14.982, p=.020), and lack of tear production ($\chi^{2}(6,893)$=22.554, p=.001). These findings suggest that while many common symptoms occur at similar frequencies across professions, certain conditions are more strongly associated with professional background, particularly distinguishing farmers from non-farmers.

**Table 6:** The Chi-square test significance results for having an eye illness among subjects with different professional engagements in agriculture. : statistically significant with p < .05, **: statistically significant with p < .01.

Do you suffer from	Level	Primarily a farmer	Secondary profession is farming	Not a farmer	$\chi^{2}(6,893)$	P
dry eyes?	Never	32 (31.1%)	77 (23.4%)	129 (28.0%)	3.857	.696
	Rarely	19 (18.4%)	66 (20.1%)	86 (18.7%)		
	Sometimes	36 (35.0%)	139 (42.2%)	180 (39.0%)		
	Always	16 (15.5%)	47 (14.3%)	66 (14.3%)		
burning eyes?	Never	26 (25.2%)	84 (25.5%)	139 (30.2%)	2.521	.641
	Rarely	70 (68.0%)	223 (67.8%)	295 (64.0%)		
	Always	7 (6.8%)	22 (6.7%)	27 (5.9%)		
red eyes?	Never	31 (30.1%)	83 (25.2%)	151 (32.8%)	22.687	.001**
	Rarely	20 (19.4%)	102 (31.0%)	155 (33.6%)		
	Sometimes	42 (40.8%)	128 (38.9%)	141 (30.6%)		
	Always	10 (9.7%)	16 (4.9%)	14 (3.0%)		
blurred vision?	Never	31 (30.1%)	84 (25.5%)	125 (27.1%)	1.058	.901
	Rarely	61 (59.2%)	206 (62.6%)	287 (62.3%)		
	Always	11 (10.7%)	39 (11.9%)	49 (10.6%)		
itchy eyes?	Never	29 (28.2%)	82 (24.9%)	108 (23.4%)	4.653	.589
	Rarely	29 (28.2%)	111 (33.7%)	134 (29.1%)		
	Sometimes	40 (38.8%)	120 (36.5%)	188 (40.8%)		
	Always	5 (4.9%)	16 (4.9%)	31 (6.7%)		
sensitivity to light?	Never	28 (27.2%)	88 (26.7%)	138 (29.9%)	3.941	.414
	Rarely	62 (60.2%)	216 (65.7%)	279 (60.5%)		
	Always	13 (12.6%)	25 (7.6%)	44 (9.5%)		
pimples inside your eyes?	Never	68 (66.0%)	259 (78.7%)	386 (83.7%)	35.629	.000**
	Rarely	10 (9.7%)	43 (13.1%)	45 (9.8%)		
	Sometimes	21 (20.4%)	24 (7.3%)	24 (5.2%)		
	Always	4 (3.9%)	3 (0.9%)	6 (1.3%)		
difficulty seeing at night?	Never	42 (40.8%)	136 (41.3%)	236 (51.2%)	14.581	.024
	Rarely	20 (19.4%)	82 (24.9%)	106 (23.0%)		
	Sometimes	36 (35.0%)	91 (27.7%)	96 (20.8%)		
	Always	5 (4.9%)	20 (6.1%)	23 (5.0%)		
headaches?	Never	22 (21.4%)	45 (13.7%)	57 (12.4%)	10.694	.030
	Rarely	62 (60.2%)	247 (75.1%)	338 (73.3%)		
	Always	19 (18.4%)	37 (11.2%)	66 (14.3%)		
severe pain in your eyes?	Never	40 (38.8%)	118 (35.9%)	188 (40.8%)	14.982	.020
	Rarely	21 (20.4%)	105 (31.9%)	136 (29.5%)		
	Sometimes	33 (32.0%)	96 (29.2%)	125 (27.1%)		
	Always	9 (8.7%)	10 (3.0%)	12 (2.6%)		
lack of tear production?	Never	45 (43.7%)	165 (50.2%)	270 (58.6%)	22.554	.001**
	Rarely	21 (20.4%)	87 (26.4%)	102 (22.1%)		
	Sometimes	28 (27.2%)	67 (20.4%)	79 (17.1%)		
	Always	9 (8.7%)	10 (3.0%)	10 (2.2%)		

Multiple linear regression was used to test if having an eye illness was explained by the significant variables in Table 6. The overall regression was statistically significant (*R*^2^ = 0.878, F(6, 889) = 1062.289, p < 0.001), see Tables 7 and 8. This indicates that the variables are able to explain 87.8% of the variance in the outcome variable. Furthermore, Multicollinearity test among the variables was highly acceptable with all VIF values lying with the range of 1.2 and 1.74.

**Table 7:** Linear regression model summary. ^a^ Predictors are the significant factors in Table 6.

Model	*R*	*R* ^*2*^	Adjusted *R*^*2*^	Std. Error of the Estimate
1	.937^a^	.878	.877	.19200

**Table 8:** Regression ANOVA results. Dependent variable is having eye illness. Predictors same as Table 7.

Model	Sum of squares	df	Mean square	F	Sig.
Regression	234.968	6	39.161	1062.289	.000
Residual	32.773	889	.037		
Total	267.741	895			

Table 9 summarizes the relationship between agricultural activities and eye-related symptoms. Most activities showed no significant differences across professions, with participants generally reporting rare occurrences of eye irritation. Specifically, pollen exposure during fruit picking ($\chi^{2}$(4,432) = 2.438, *p* = 0.295), soil particles when digging ($\chi^{2}$(4,432) = 3.530, *p* = 0.171), pesticide spraying ($\chi^{2}$(4,432) = 1.187, *p* = 0.552), and digging-related eye injuries ($\chi^{2}$(4,432) = 3.263, *p* = 0.196) showed no significant associations. Fruit-related eye allergies showed a near-significant trend ($\chi^{2}$(4,432) = 5.960, *p* = 0.051). By contrast, tree pruning ($\chi^{2}$(4,432) = 11.994, *p* = 0.002) and crop watering ($\chi^{2}$(4,432) = 7.758, *p* = 0.021) revealed significant differences, with agricultural workers more likely to report eye injuries and non-farmers reporting fewer problems. Overall, the results suggest that while most agricultural activities pose limited eye health risks, specific tasks such as pruning and watering are more strongly linked to eye problems.

**Table 9:** The Chi-square test significance results for the effect of agricultural activities on eye health among subjects with different professional engagements in agriculture. : statistically significant with p < .05, **: statistically significant with p < .01

Question	Level	Primarily a farmer	Secondary profession is farming	Not a farmer	$\chi^{2}(4,432)$	P
When picking fruits, do you get pollen in your eyes?	Never	14 (13.6%)	38 (11.6%)	-	2.438	.295
	Rarely	70 (68.0%)	248 (75.4%)	-		
	Always	19 (18.4%)	43 (13.1%)	-		
When digging to plant trees, do you risk getting soil particles into your eyes?	Never	6 (5.8%)	31 (9.4%)	-	3.530	.171
	Rarely	78 (75.7%)	258 (78.4%)	-		
	Always	19 (18.4%)	40 (12.2%)	-		
Do you suffer from eye allergy to any fruit?	Never	28 (27.2%)	128 (38.9%)	-	5.960	.051
	Rarely	61 (59.2%)	174 (52.9%)	-		
	Always	14 (13.6%)	27 (8.2%)	-		
When spraying pesticides, do you suffer from eye injuries?	Never	20 (19.4%)	68 (20.7%)	-	1.187	.552
	Rarely	68 (66.0%)	226 (68.7%)	-		
	Always	15 (14.6%)	35 (10.6%)	-		
When digging the soil, do you suffer from eye injuries?	Never	21 (20.4%)	70 (21.3%)	-	3.263	.196
	Rarely	69 (67.0%)	236 (71.7%)	-		
	Always	13 (12.6%)	23 (7.0%)	-		
When pruning trees, do you suffer from eye injuries?	Never	27 (26.2%)	93 (28.3%)	-	11.994	.002**
	Rarely	60 (58.3%)	219 (66.6%)	-		
	Always	16 (15.5%)	17 (5.2%)	-		
When watering the field, do you suffer from eye problems?	Never	43 (41.7%)	165 (50.2%)	-	7.758	.021
	Rarely	50 (48.5%)	153 (46.5%)	-		
	Always	10 (9.7%)	11 (3.3%)	-		

Multiple linear regression was used to test if the effect of agricultural activities on eye health was explained by the significant variables in Table 9. The overall regression was statistically significant (*R*^2^ = 0.638, F(2, 432) = 380.441, p < 0.001), see Tables 10 and 11. This indicates that the variables are able to explain 63.8% of the variance in the outcome variable. Furthermore, Multicollinearity test among the variables was highly acceptable with a VIF value of 1.394 for both significant variables.

**Table 10:** Linear regression model summary. ^a^ Predictors are the significant factors in Table 9.

Model	*R*	*R* ^*2*^	Adjusted *R*^*2*^	Std. Error of the Estimate
1	.799^a^	.638	.636	.31840

**Table 11:** Regression ANOVA results. Dependent variable is effect of agricultural activities on eye health. Predictors same as Table 10.

Model	Sum of squares	df	Mean square	F	Sig.
Regression	77.138	2	38.569	380.441	.000
Residual	43.796	432	.101		
Total	120.934	434			

Table 12 summarizes eyewear and eye protection usage. Prescription glasses were never used by 49.8% of respondents, with no significant differences across professions ($\chi^{2}$(6,893) = 8.780, *p* = 0.186). Similarly, sunglasses showed no significant variation ($\chi^{2}$(6,893) = 8.910, *p* = 0.179), though non-farmers reported slightly higher regular use. Eye protection, however, revealed significant differences: 53.9% reported never using it, with the highest rate among non-farmers (61.4%) ($\chi^{2}$(6,893) = 28.139, *p* = 0.000). When asked specifically about agricultural practices, 24.8% never used protection, with agricultural workers showing the highest non-use (27.4%), while non-farmers reported more frequent use ($\chi^{2}$(6,432) = 14.319, *p* = 0.003). Overall, while prescription glasses and sunglasses use do not differ by profession, agricultural workers are less likely to use protective equipment, highlighting the need for greater awareness and adoption of safety measures.

**Table 12:** The Chi-square test significance results for the use of protective equipment among subjects with different professional engagements in agriculture. N=893 except for the last question, N=432. **: statistically significant with p < .01.

Do you use	Level	Primarily a farmer	Secondary profession is farming	Not a farmer	$\chi^{2}(6)$	P
prescription glasses?	Never	53 (51.5%)	147 (44.7%)	245 (53.1%)	8.780	.186
	Rarely	9 (8.7%)	40 (12.2%)	34 (7.4%)		
	Sometimes	23 (22.3%)	85 (25.8%)	102 (22.1%)		
	Always	18 (17.5%)	57 (17.3%)	80 (17.4%)		
sunglasses?	Never	18 (17.5%)	88 (26.7%)	140 (30.4%)	8.910	.179
	Rarely	21 (20.4%)	65 (19.8%)	84 (18.2%)		
	Sometimes	47 (45.6%)	140 (42.6%)	178 (38.6%)		
	Always	17 (16.5%)	36 (10.9%)	59 (12.8%)		
eye protection?	Never	47 (45.6%)	151 (45.9%)	283 (61.4%)	28.139	.000**
	Rarely	16 (15.5%)	74 (22.5%)	62 (13.4%)		
	Sometimes	23 (22.3%)	72 (21.9%)	76 (16.5%)		
	Always	17 (16.5%)	32 (9.7%)	40 (8.7%)		
eye protection while practicing agriculture?	Never	17 (16.5%)	90 (27.4%)	-	14.319	.003**
	Rarely	19 (18.4%)	80 (24.3%)	-		
	Sometimes	31 (30.1%)	98 (29.8%)	-		
	Always	36 (35.0%)	61 (18.5%)	-		

Multiple linear regression was used to test if the use of protective equipment was explained by the significant variables in Table 12. The overall regression was statistically significant (*R*^2^ = 0.712, F(2, 432) = 534.528, p < 0.001), see Tables 13 and 14. This indicates that the variables are able to explain 71.2% of the variance in the outcome variable. Furthermore, Multicollinearity test among the variables was highly acceptable with a VIF value of 1.017 for both significant variables.

**Table 13:** Linear regression model summary. ^a^ Predictors: (Constant), Time you work, I visit an eye doctor every, Income, Gender, Age, Nature of work, Work sector, Number of working days per week, Educational level.

Model	*R*	*R* ^*2*^	Adjusted *R*^*2*^	Std. Error of the Estimate
1	.844^a^	.712	.711	.38126

**Table 14:** Regression ANOVA results. Dependent variable is use of protective equipment. Predictors same as Table 13.

Model	Sum of squares	df	Mean square	F	Sig.
Regression	155.397	2	77.699	534.528	.000
Residual	62.795	432	.145		
Total	218.193	434			

Table 15 presents one-way ANOVA results on eye health-related variables across professions. Each variable was generated using the sum of answers to the questions in the corresponding table. Farmers, particularly those with agriculture as their main job, reported stronger perceptions of climate and agricultural work affecting their eye health and higher use of protective equipment. Weather impact differed significantly (F(2,890) = 5.916, *p* = 0.003), with main-profession farmers reporting the highest mean (2.20), followed by secondary farmers (2.12) and non-farmers (2.03). Perceptions of overall eye health issues were slightly higher among farmers (main = 2.05, secondary = 1.98) than non-farmers (1.92), though not significant (F(2,890) = 2.849, *p* = 0.058). Agricultural work’s impact on eye health was significant (F(1,430) = 7.400, *p* = 0.007), with main-profession farmers (2.07) reporting greater effects than secondary farmers (1.91). Eye protection use also differed significantly (F(1,430) = 5.041, *p* = 0.025), with main-profession farmers (2.40) more likely to adopt protection than secondary farmers (2.22).

**Table 15:** The one-way ANOVA test results. : statistically significant with p < .05, **: statistically significant with p <.01.

Variable	Classification	N	Mean	SD	Test	p
Weather impact on eye health.	Primarily a farmer	103	2.20	0.62	F(2,890)=5.916	.003**
	Secondary profession is farming	329	2.12	0.49		
	Not a farmer	461	2.03	0.51		
Having eye illness	Primarily a farmer	103	2.05	0.67	F(2,890)=2.849	.058
	Secondary profession is farming	329	1.98	0.53		
	Not a farmer	461	1.92	0.53		
The impact of agricultural work on eye health	Primarily a farmer	103	2.07	0.65	F(1,430)=7.400	.007**
	Secondary profession is farming	329	1.91	0.48		
Use of eye protective equipment	Primarily a farmer	103	2.40	0.77	F(1,430)=5.041	.025
	Secondary profession is farming	329	2.22	0.69		

Table 16 shows the LSD pair-wise comparisons. The table reveals that the difference between the “primarily a farmer” group and the “secondary farmers” group is not statistically significant, with a mean difference of 0.07979, while a statistically significant difference exists between “primarily a farmer” and “not a farmer” groups (mean difference = 0.16952), indicating that those whose main profession is farming suffer a greater impact on eye health. Comparisons between “secondary farmers” and “not a farmer” also show a small mean difference of 0.08972, and it is statistically significant. Overall, the results suggest that those in primary/secondary agricultural roles are more concerned about eye health than non-farmers, while the differences between primarily farming professionals and secondary farmers are less pronounced.

**Table 16:** LSD results for the climate impact on eye health based on subject’s profession. **: statistically significant difference with *p*<.01

	Mean	Primarily a farmer	Secondary profession is farming	Not a farmer
Primarily a farmer	2.20	—		
Secondary profession is farming	2.12	.07979	—	
Not a farmer	2.03	.16952**	.08972**	—

## Discussion and conclusion

Studying the effect of weather/climate conditions and farming activities on farmers’ eye health is important due to the variety of risks posed by environmental and occupational factors, which are exacerbated by changing climatic conditions. Increased ultraviolet (UV) radiation, a consequence of ozone layer depletion and climate change, heightens the risk of cataracts, pterygium, and photokeratitis among farmers who spend prolonged hours outdoors [21,22]. Furthermore, farming activities such as plowing and harvesting generate dust and particulate matter, which can cause chronic eye irritation, conjunctivitis, and dry eye syndrome, with climate change intensifying these risks through more frequent droughts and dust storms [23]. Exposure to agricultural chemicals like pesticides and herbicides further compounds the problem, leading to chemical burns and ocular surface disorders, with climate change potentially altering the dispersal and volatility of these substances [24]. Rising temperatures also contribute to heat stress, reducing tear production and exacerbating dry eye disease, particularly among farmers working in hot environments [25]. Moreover, climate change influences the spread of vector-borne diseases such as trachoma, a leading cause of preventable blindness, particularly in rural farming communities [26]. The economic and social implications of poor eye health among farmers are significant, as vision impairment can reduce productivity, increase healthcare costs, and threaten food security, especially in developing regions with limited access to eye care [27]. Addressing these challenges requires adaptive strategies, including protective eyewear, improved farming practices, and community-based health education, to mitigate the compounded effects of climate change and occupational hazards on farmers’ eye health [28].

The present findings that primary farmers reported significantly higher eye problems in cold, rainy, and dusty weather highlight the unique occupational vulnerabilities of individuals whose main livelihood depends on farming. Cold weather can destabilize the tear film, lower ambient humidity, and expose farmers to wind, thereby exacerbating symptoms such as ocular dryness, irritation, and discomfort, as confirmed by studies showing that cold weather is a common aggravating factor in dry eye conditions [29]. Rainy or wet weather, while increasing humidity, creates favorable conditions for microbial growth and facilitates the entry of soil, debris, and infectious agents into the eye, predisposing to conjunctivitis, fungal keratitis, and other ocular infections, which have been reported to rise during monsoon or wet seasons [30]. Dust exposure represents one of the most documented occupational hazards for farmers, as organic and inorganic dust particles mechanically irritate the conjunctiva and cornea; consistent with this, a Norwegian study demonstrated higher rates of eye irritation during dusty agricultural tasks such as grain handling and livestock feeding [31]. Taken together, the elevated mean scores among primary farmers reflect both greater duration and intensity of exposure to environmental stressors and the lack of widespread use of protective equipment in these communities.

The higher perception of eye illness symptoms such as red eyes and pain, pimples inside the eyes, headaches, and lack of tear production among primary farmers compared to secondary farmers and non-farmers indicate the cumulative effect of prolonged and direct agricultural exposure on ocular health. Red eyes and ocular pain often reflect conjunctival or corneal inflammation, which can arise from chronic exposure to dust, pesticides, ultraviolet radiation, and other irritants common in farming environments. The occurrence of “pimples inside eyes,” likely referring to chalazia or styes, is consistent with blocked meibomian glands or recurrent bacterial infections, conditions that are more prevalent in environments with poor hygiene and continuous exposure to organic dust [32]. Headaches, on the other hand, may be secondary to uncorrected refractive errors exacerbated by eye strain, or linked to ocular surface irritation from constant environmental stressors [33]. The lack of tear production is indicative of dry eye disease, a condition strongly associated with chronic outdoor work, excessive UV exposure, and wind, as documented in rural populations where farming is a dominant occupation [34].

Agricultural workers engaged in diverse tasks such as picking fruit and pollen exposure, digging and soil contact, handling pesticides, pruning vegetation, and watering crops are subject to multiple overlapping risk factors for ocular irritation, allergic reactions, and more serious eye injuries. Pollen and airborne soil or dust from digging can trigger allergic conjunctivitis, mechanical irritation and foreign body entry into the eye; pruning and brushing against branches increase risk of abrasion or penetrating trauma. Pesticide use compounds this by introducing chemical irritants and toxins that stimulate symptoms such as burning, watering, redness, and possibly contribute to long-term damage such as retinal degeneration. Empirical studies support these associations. For example, farmers in Thailand reported high rates of itchy eyes, burning and conjunctivitis linked with pesticide exposure (26.3%) when using insecticides, fungicides and rodenticides [35]. In Ghana, watermelon farmers similarly reported burning sensation and watering of eyes as common health risk symptoms in relation to pesticide use and lack of protective equipment [5]. An Indian study across two states found that agricultural tasks such as weed removal, harvesting, and pesticide spraying were associated with ocular morbidities and foreign body injuries, and that the use of safety eyewear improved comfort and reduced exposure to hazards including dust and flying particles [36]. Thus, when comparing primary farmers, who perform these tasks more intensively, to secondary or non-farmers, it is plausible and supported by the literature that primary farmers show higher mean scores for eye illness symptoms.

The last variable, use of eye protective equipment, assessed both general use and use specifically during agricultural activities. Results show that farmers are more inclined to wear protective equipment compared to non-farmers, both in general and while practicing agriculture. This not only highlights a positive trend toward preventive behavior among farmers but also reinforces the quality and reliability of the self-reported data, as the pattern aligns with the higher exposure risks identified in this group. Furthermore, evidence from different regions shows that farmers are generally more inclined to use eye protection, especially when engaged in high-risk agricultural tasks, which supports the reliability of our data. For instance, intervention studies among citrus harvesters in Florida demonstrated that providing well-designed protective eyewear and peer support increased usage from less than 2% to nearly 37% across seasons [37], while similar findings in India confirmed that refractive safety eyewear improved comfort, reduced dust exposure, and lowered ocular morbidity among farmers [17,38]. Nonetheless, despite these benefits, barriers such as discomfort, poor fit, or lack of awareness remain common, leading to inconsistent use [38]. A two-state study in India further emphasized that agriculture workers face high risks of ocular injury but often underutilize protective equipment [36]. Similarly, Latino farmworkers in North Carolina reported very low baseline use (8.3%), but usage increased when employers mandated or supplied protective gear [39].

This study has several limitations that should be acknowledged. First, the sample included a disproportionate number of farmers compared to other occupational groups. While this reflects the demographic reality of the study community, where farming, whether as a primary or secondary activity, is common, it may limit the generalizability of the findings to populations with different occupational distributions and subsequently affect the validity of the statistical analysis. Second, a substantial proportion of participants engaged in farming as a secondary profession while their primary occupation was in government or office-based work. Because these primary professions were not systematically stratified in the analysis, potential confounding cannot be excluded, particularly since occupational exposures in the primary profession may have contributed to health outcomes independent of farming. Third, some of the conditions discussed, such as dry eye, require clinical evaluation and formal diagnostic criteria for confirmation. In this study, no clinical diagnoses were performed; rather, symptoms were self-reported. As such, these findings should be interpreted with caution, and future studies employing clinical assessment are warranted to validate these observations.

Despite these limitations, the study draws attention to an important but often overlooked issue: the eye health of farmers in rural communities, particularly in regions with limited healthcare infrastructure, minimal use of protective measures, and little prior research. Paying closer attention to the occupational health of farmers in such settings is critical, as they are disproportionately exposed to environmental and occupational hazards yet receive less medical care and protective guidance. Addressing this gap not only strengthens public health initiatives in under-studied populations but also supports the development of targeted interventions (e.g., education on protective equipment use, and improved healthcare access) that can significantly reduce preventable eye problems among agricultural workers.

Given the perceived impact of farming on eye health, several recommendations can help mitigate risks and promote protective measures. Increasing awareness of climate-related eye health risks is not a luxury. Farmers should be educated through awareness campaigns about the harmful effects of prolonged sun exposure, airborne pollutants, and agricultural chemicals. Moreover, enhancing the use of protective eyewear is important since studies in the literature have shown that proper eye protection can reduce the incidence of work-related eye injuries [40]. Policies should mandate the use of safety goggles, wrap-around sunglasses, and other personal protective equipment (PPE) when handling soil, pesticides, and crops. Reducing exposure to agricultural hazards can further minimize eye health risks, as studies have shown that pesticide exposure significantly increases the likelihood of ocular toxicity and irritation [14]. This brings into the fore the need for farmers to wear masks and goggles during activities such as spraying pesticides or pruning trees. Regular eye examinations should also be promoted to detect and address vision problems early, particularly among agricultural workers frequently exposed to dust and chemicals, as occupational exposure to particulate matter has been associated with increased eye health complications [36]. Healthcare providers and governments should facilitate mobile eye clinics in rural farming communities to ensure accessible checkups. Improving agricultural safety training programs is another vital strategy, as structured training significantly enhances compliance with eye protection recommendations [41]. Farmers should be educated on safe handling techniques for chemicals and soil to minimize direct contact with hazardous materials. Furthermore, encouraging the adoption of safer farming practices through mechanization and precision agriculture can help reduce direct exposure to harmful elements, as modernized techniques lower the risk of eye injuries by limiting manual interactions with dust and chemicals [17].

Policymakers should support farmers in transitioning to these safer technologies through subsidies or training programs. Alternatives such as organic and nature-based farming systems offer viable pathways to minimize chemical dependency while maintaining soil fertility and crop yield [42]. Similarly, integrated pest management and the use of biopesticides, derived from natural organisms or substances, can effectively control pests with lower ecological and health impacts [43,44]. Encouraging the adoption of these practices through awareness campaigns, training programs, and policy support may help safeguard both farmer well-being and environmental sustainability.

## Supporting information

S1 File Research data. The clean anonymized data entered by the respondents.
(XLSX)
